# Efficacy and safety of the thiotepa–busulfan conditioning regimen as for autologous stem cell transplantation in relapsed/refractory systemic diffuse large B cell lymphoma: a single-center retrospective study

**DOI:** 10.1007/s12185-025-03946-w

**Published:** 2025-02-12

**Authors:** Katsuhiro Io, Kenichi Nagai, Bunta Kakihara, Kiyotaka Izumi, Tomoya Kitagawa

**Affiliations:** Department of Hematology, Kansai Electric Power Hospital, 2-1-7 Fukushima, Fukushima-ku, Osaka 553-0003 Japan

**Keywords:** Relapsed/refractory diffuse large B cell lymphoma, Autologous stem cell transplantation, Thiotepa–busulfan conditioning regimen

## Abstract

High-dose chemotherapy followed by autologous stem cell transplantation (ASCT) is a standard treatment for relapsed diffuse large B cell lymphoma (DLBCL). The BEAM regimen is widely used, but the MEAM regimen is more common in Japan due to the unavailability of carmustine. This retrospective analysis evaluated the efficacy of the thiotepa and busulfan (TT/BU) regimen compared with other regimens in 27 patients with systemic DLBCL who underwent ASCT at our institution from December 2013 to March 2022. Fourteen patients received the TT/BU regimen, while 13 received alternative regimens. The TT/BU regimen demonstrated superior progression-free survival (PFS) and overall survival (OS) compared to other regimens, with a 3 year PFS of 84.4% and OS of 91.7%. The TT/BU group also had fewer severe adverse events, particularly regarding renal function. Our findings suggest that the TT/BU regimen is a well-tolerated and effective alternative for relapsed/refractory DLBCL and provide valuable insights for future treatment strategies.

**Supplementary Information:**

The online version contains supplementary material available at 10.1007/s12185-025-03946-w.

## Introduction

High-dose chemotherapy followed by autologous stem cell transplantation (ASCT) is an established treatment for relapsed diffuse large B cell lymphoma (DLBCL). [1–4] The BEAM regimen (carmustine, etoposide, cytarabine, melphalan) is widely used worldwide as the standard high-dose therapy (HDT) prior to ASCT. [5] In Japan, the MEAM regimen (a modified version of BEAM using ranimustine) is the most commonly employed due to the unavailability of carmustine. [6, 7] Other commonly used regimens for ASCT include BeEAM (bendamustine, etoposide, cytarabine, melphalan), [8] BUMEL (busulfan, melphalan), [9] and BuCyE (busulfan, cyclophosphamide, etoposide). [10]Thiotepa, an alkylating agent, is approved for high-dose preparation before ASCT for lymphoma and is particularly effective in crossing the blood–brain barrier, making it a regular component of HDT followed by ASCT for primary central nervous system lymphoma (PCNSL). [11–14]Some studies have indicated that thiotepa-based regimens for ASCT in systemic lymphoma are comparable to conventional regimens like BEAM. [15, 16] However, there is limited clinical-level information available on the TT/BU (thiotepa, busulfan) regimen, which is utilized in Japan.

To evaluate the efficacy of the TT/BU regimen in comparison to other regimens for DLBCL, we conducted a retrospective analysis of 23 patients with systemic DLBCL who underwent ASCT at our institution from December 2013 to March 2022.

## Methods

Patient selection: this study included 27 patients who underwent autologous peripheral blood stem cell transplantation for diffuse large B cell lymphoma (DLBCL) at Kansai Electric Power Hospital from December 2013 to December 2022. Patients were followed up until June 2024. Patients with primary central nervous system lymphoma were excluded, while those with DLBCL and central nervous system relapse were included. It was at the discretion of the attending physician which regimen to use and whether to use rituximab in combination. Clinical data and follow-up information were obtained from the patients’ medical records. Performance status was assessed using the Eastern Cooperative Oncology Group scale. The study protocol was approved by the Institutional Review Board of Kansai Electric Power Hospital in accordance with the Declaration of Helsinki.

Treatments: the TT/BU regimen, which was covered by insurance in Japan, consisted of thiotepa at a dosage of 5 mg/kg from day 4 to day 3, and busulfan at a dosage of 3.2 mg/kg from day 8 to day 5. In our institute, the MEAM regimen included ranimustine at a dosage of 300 mg/m^2^ on day 7, etoposide at a dosage of 200 mg/m^2^ from day 6 to day 3, cytarabine at a dosage of 200 mg/m^2^ twice daily from day 6 to day 3, and melphalan at a dosage of 140 mg/m^2^ on day 2. The BeEAM regimen consisted of bendamustine at a dosage of 200 mg/m^2^ from day 8 to day 7, etoposide at a dosage of 100 mg/m^2^ twice daily from day 6 to day 3, cytarabine at a dosage of 200 mg/m^2^ twice daily from day 6 to day 3, and melphalan at a dosage of 140 mg/m^2^ on day 2. The BUMEL regimen included busulfan at a dosage of 130 mg/m^2^ from day 8 to day 5 and melphalan at a dosage of 70 mg/m^2^ from day 3 to day 2. The BuCyE regimen consisted of busulfan at a dosage of 3.2 mg/kg from day 7 to day 5, etoposide at a dosage of 200 mg/m^2^ twice daily from day 5 to day 4, cyclophosphamide at a dosage of 50 mg/kg from day 3 to day 2.

Statistical analysis: the main objective of this study is to compare Overall Survival (OS) and Progression-Free Survival (PFS) of patients who underwent ASCT with the TT/BU regimen to those who received other regimens. OS was calculated from the date of stem cell transplantation to the date of death. PFS was measured from the date of stem cell transplantation to the occurrence of relapse, progression, or death from any cause.

To compare various parameters between the TT/BU group and the other regimens group, Fisher’s exact test was employed. Actuarial survival analysis was conducted using the Kaplan–Meier method, and the resulting survival curves were compared using the log-rank test. All reported *P* values are two-sided, and *P* values less than 0.05 were considered statistically significant.

Statistical analysis was performed using EZR (Saitama Medical Center, Jichi Medical University), which is a graphical user interface for R (The R Foundation for Statistical Computing) [17]. EZR is a modified version of the *R* Commander that incorporates commonly used biostatistical functions.

## Results

Between December 2013 and March 2022, a total of 27 patients underwent autologous peripheral blood stem cell transplantation at Kansai Electric Power Hospital. Of these patients, 14 underwent pre-conditioning with the TT/BU regimen, while the remaining 13 received alternative regimens like MEAM. Baseline characteristics at the time of HDT-ASCT are summarized in Table 1. No statistically significant differences were observed between the two regimen groups in terms of age, gender, degree of remission at the time of transplantation, disease status, or CNS invasion. Notably, the use of rituximab as concurrent therapy was significantly less frequent in the TT/BU regimen group.

**Table 1:** Patient Characteristics at time auto stem cell transplantation

Characteristic	TT/BU regimen *n* = 14	Other regimens *n* = 13	*p* value
Age at ASCT, yr, median (range)	62(47–70)	64(48–76)	0.733
Sex, n			
Female	7	2	0.103
male	7	11	
Disease status at HDT-ASCT, n			
CR	14	10	0.098
PR	0	3	
SD/PD	0	0	
HDT regimen prior to ASCT			
TT/BU	14	0	< 0.001
MEAM	0	10	
BeEAM	0	1	
BUMEL	0	1	
BuCyE	0	1	
Rituximab addition in HDT, n			
Yes	1	9	0.001
No	13	4	
Disease status, n			
Upfront	5	1	0.165
Relapse/Refractory	9	12	
Primary refractory	3	6	
Relapse within one year	4	1	
Relapse after more than one year	2	5	
CNS invasion, n			
Yes	2	1	1
No	12	12	
Number of infused CD34-positive cells, median (range)	3.63 × 10 ^6^ (2.23–7.16)	2.30 × 10 ^6^ (1.50–7.20)	0.058

*ASCT* autologous stem cell transplantation, *HDT* high-dose chemotherapy, *CNS* central nervous system, *TT* thiotepa, *BU* busulfan

Each group contained seven cases of relapse or refractory within one year following first-line treatment, with no significant differences observed. Furthermore, there were no disparities in the number of infused stem cells. Although the median observation period was longer in the TT/BU regimen group, alternative regimens group exhibited a wide range of observation periods, ranging from short term to long term. Ultimately, no statistically significant differences were found between the two groups.

Table 2 provides a summary of the adverse effects observed in both groups. In both regimen groups, diarrhea and nausea were prevalent, with approximately 30% of cases classified as grade 3 or higher though no statistically significant differences were found. There were no significant differences between the groups regarding bleeding, edema, and infections. However, with regard to renal impairment, the TT/BU group exhibited a statistically significantly lower incidence. Delayed-onset thrombocytopenia was observed not only in the TT/BU regimen group but also in the alternative regimens group.

**Table 2:** Summary of the adverse effects observed in TT/BU regimen group and other regimens group

Adverse events	TT/BU regimen *n* = 14	Other regimens *n* = 13	*P* value
Febrile neutropenia			
Grades 1–2	14(100)	13(100)	1
Grades 3–4	14(100)	13(100)	
Diarrhea (%)			
Grades 1–2	3 (21.4)	1 (7.7)	0.656
Grades 3–4	4 (28.6)	3 (23.1)	
Nausea (%)			
Grades 1–2	5 (35.7)	5 (38.5)	0.551
Grades 3–4	5 (35.7)	2 (15.4)	
Bleeding (%)			
Grades 1–2	0 (0.0)	1 (7.7)	0.222
Grades 3–4	0 (0.0)	1 (7.7)	
Edema (%)			
Grades 1–2	0 (0.0)	0 (0.0)	0.222
Grades 3–4	0 (0.0)	2 (15.4)	
Infection without FN (%)			
Grades 1–2	1 (7.1)	2 (15.4)	0.596
Grades 3–4	0 (0.0)	0 (0.0)	
Renal Impairment (%)			
Grades 1–2	0 (0.0)	2 (15.4)	0.041
Grades 3–4	0 (0.0)	2 (15.4)	
SIADH (%)			
Grades 1–2	1 (7.1)	0 (0.0)	1
Grades 3–4	0 (0.0)	0 (0.0)	
TMA (%)			
Grades 1–2	0 (0.0)	0 (0.0)	0.481
Grades 3–4	0 (0.0)	1 (7.7)	
VOD (%)			
Grades 1–2	0 (0.0)	0 (0.0)	NA
Grades 3–4	0 (0.0)	0 (0.0)	
Dysuria (%)			
Grades 1–2	0 (0.0)	1 (7.7)	0.481
Grades 3–4	0 (0.0)	0 (0.0)	
Delayed thrombocytopenia (%)			
Yes	5 (35.7)	4 (30.8)	1
No	9 (64.3)	9 (69.2)	

*FN* febrile neutropenia, *SIADH* Syndrome of inappropriate secretion of Antidiuretic Hormone, *TMA* thrombotic microangiopathy, *VOD* venoocclusive disease, *TT* thiotepa, *BU* busulfan

**Fig. 1:**
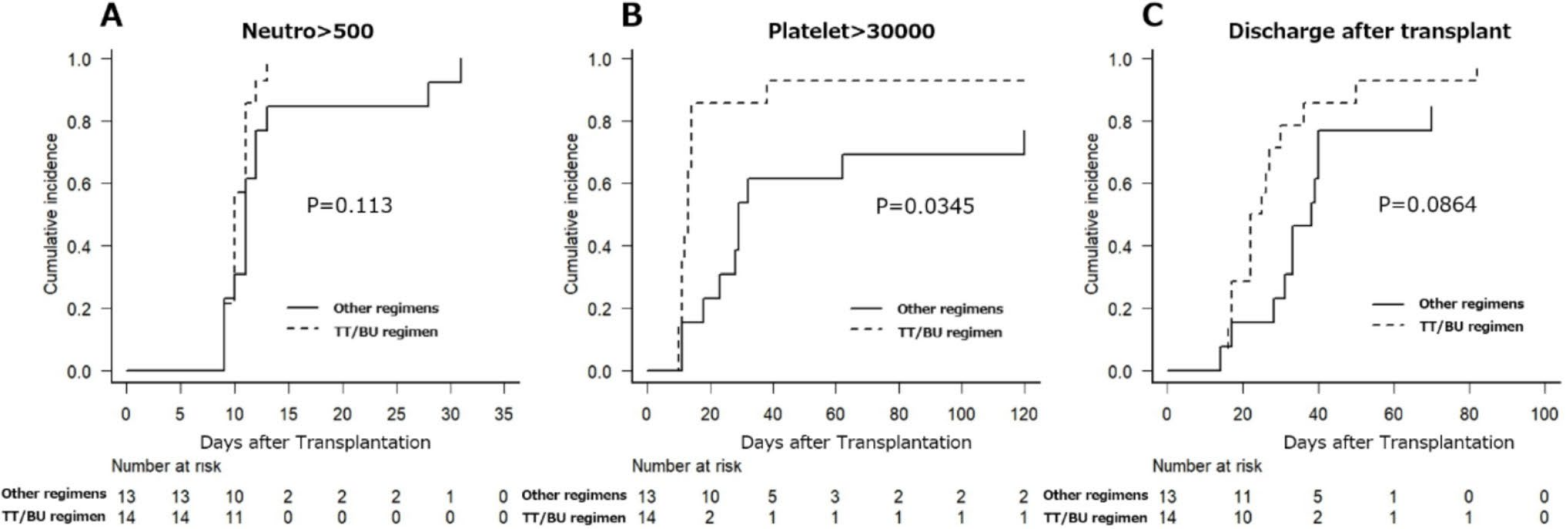
Cumulative achievement rate of neutrophil > 500/μL (**A**) and Platelet > 30,000/μL (**B**) after autologous hematopoietic stem cell transplantation according to high-dose chemotherapy regimens. Cumulative discharge rate after transplantation (**C**) after transplantation. *Neutro* neutrophils; *TT* thiotepa, *BU* busulfan, *p* p value

The time required for the absolute neutrophil count to reach 500/μL did not show a significant difference between the TT/BU regimen group and the other regimen groups, with medians of 10 and 11 days, respectively(Fig. 1A). However, the time taken for the platelet count to reach 30,000/μL was significantly shorter in the TT/BU group, with a median of 13 days (range 11-NA), compared to a median of 29 days (range 23-NA) in the other regimen groups. (Fig. 1B) This difference was statistically significant (*p* = 0.0345). While not reaching statistical significance, there was a trend toward a shorter duration from transplant to discharge in the TT/BU group, with a median of 23.5 days (range 17-NA), compared to a median of 38 days (range 31-NA) in the other regimen groups. (Fig. 1C).

**Fig. 2:**
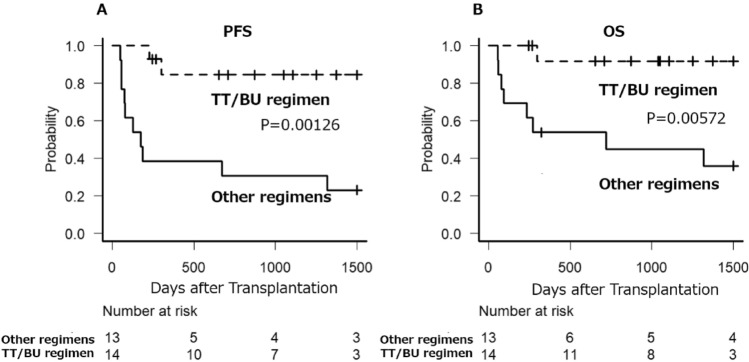
Progression-free survival (**A**) and Overall survival (**B**) after autologous hematopoietic stem cell transplantation according to high-dose chemotherapy regimens. *TT* thiotepa, *BU* busulfan, *p* p value

The PFS for the TT/BU group exhibited a median that was not applicable (NA), with a 95% confidence interval of NA-NA. The 3 year PFS significantly extended to 84.4% (95% confidence interval: 50.4–95.9) compared to the other regimen group, which had a median of 175 days (95% confidence interval: 56–1318) and a 3 year PFS of 30.8% (95% confidence interval: 9.5–55.4) (*p* = 0.00126). (Fig. 2A).

Furthermore, with regard to OS, the TT/BU group demonstrated a median that was not applicable (NA), with a 95% confidence interval of NA-NA. The 3 year OS was superior at 91.7% (95% confidence interval: 53.9–98.8) compared to the other regimen group, which had a median of 719 days (95% confidence interval: 76-NA) and a 3 year OS of 44.9% (95% confidence interval: 17.7–69) (*p* = 0.00572). (Fig. 2B).

Even among patients in complete remission at the time of transplantation, the TT/BU regimen demonstrated significantly superior PFS. The median PFS was not reached (95% confidence interval: NA-NA), with a 3 year PFS of 84.4% (95% confidence interval: 50.4–95.9), compared to a median PFS of 428 days (95% confidence interval: 56-NA) and a 3 year PFS of 40.0% (95% confidence interval: 12.3–67.0) in the alternative regimen group (*p* = 0.00833) (Supplementary Fig. 1A). Similarly, overall OS was significantly better in the TT/BU group, with the median OS not reached (95% confidence interval: NA-NA) and a 3 year OS of 91.7% (95% confidence interval: 53.9–98.8), compared to a median OS of 1318 days (95% confidence interval: 56-NA) and a 3 year OS of 58.3% (95% confidence interval: 23.0–82.1) in the alternative regimen group (*p* = 0.0398) (Supplementary Fig. 1B).

Among patients with refractory disease or relapse within one year following first-line treatment, the TT/BU regimen also demonstrated significantly superior PFS. The median PFS was not reached (95% confidence interval: 225-NA), with a 3 year PFS of 64.3% (95% confidence interval: 15.1–90.2), compared to a median PFS of 175 days (95% confidence interval: 48–1348) and a 3 year PFS rate of 28.6% (95% confidence interval: 4.1–61.2) in the alternative regimen group (*p* = 0.0318) (Supplementary Fig. 2A). Regarding OS, the TT/BU group exhibited a trend toward improved outcomes, with the median OS not reached (95% confidence interval: 296-NA) and a 3 year OS of 80.0% (95% confidence interval: 20.4–96.9), compared to a median OS of 234 days (95% confidence interval: 56-NA) and a 3 year OS of 42.9% (95% confidence interval: 9.8–73.4) in the alternative regimen group although the difference did not reach statistical significance (*p* = 0.0577) (Supplementary Fig. 2B).

## Discussion

High-dose chemotherapy followed by ASCT is a standard treatment for relapsed and refractory DLBCL in young patients. [1–4] Various conditioning regimens are employed prior to ASCT, with the BEAM regimen being widely used and often replaced by the MEAM regimen in Japan. [5–7] However, there is a need for alternative agents to replace BEAM or MEAM due to the potential side effects and supply issues associated with melphalan and ranimustine.

Although numerous reports demonstrate the efficacy of conditioning regimens that include thiotepa, most focus on central nervous system lymphomas, [11–14] with limited comparisons to other conditioning regimens in systemic lymphoma. [15, 16] Furthermore, reports indicate that thiotepa-containing regimens usually involve combinations with three or more agents, such as busulfan, cyclophosphamide, or melphalan. [11–16] Studies investigating the effectiveness and toxicity of the thiotepa and busulfan (TT/BU) regimen, which is approved for insurance coverage in Japan, in the context of systemic DLBCL are limited [18].

Therefore, although this is a retrospective analysis conducted at a single institution, our study provides valuable insights into the efficacy and the toxicity of the two-agent combination of thiotepa and busulfan for ASCT in systemic DLBCL. This analysis contributes valuable information for future lymphoma treatment strategies.

In primary central nervous system DLBCL, two-agent regimens containing thiotepa are associated with fewer adverse events and treatment-related deaths compared to regimens incorporating three or more agents, including thiotepa. [19–23] Similar toxicities are expected in systemic lymphoma. Our analysis found that the TT/BU regimen had minimal severe adverse events. Compared to previous regimens, the toxicity of the TT/BU regimen, particularly with respect to renal function, appears to be either equivalent or lower. These findings suggest that the TT/BU regimen has a favorable safety profile and can be a well-tolerated therapeutic option for relapsed/refractory (R/R) DLBCL. In a prior Japanese report, delayed-onset thrombocytopenia was identified as a side effect of the TT/BU regimen. [24] However, our facility’s analysis showed that delayed-onset thrombocytopenia occurred with the TT/BU regimen, but its incidence was not significantly higher compared to preceding treatment protocols. This difference from previous findings may be due to the limited use of rituximab with the conditioning regimen for ASCT within the TT/BU regimen cohort [25].

Our study is a single-center retrospective analysis focusing on the primary endpoints of overall survival and progression-free survival. The results demonstrate the superiority of the TT/BU regimen over prior treatment protocols and this report represents the first evidence of such efficacy observed with a two-drug regimen including thiotepa for systemic DLBCL. There were no significant differences between the TT/BU group and the previous regimen group in patient background factors likely to influence treatment outcomes, such as rates of relapse within one year or achievement of remission at the time of transplantation. However, it is important to note that, although the difference was not statistically significant, all patients in the TT/BU regimen group underwent transplantation in a CR state, whereas three patients in the alternative regimen group were transplanted in PR. To address this potential confounding factor, we performed a subgroup analysis restricted to patients in CR at the time of transplantation. In this subset, the TT/BU regimen still demonstrated significantly superior PFS and OS compared to the alternative regimen group.

In recent years, CAR T therapy has become a viable option for treating DLBCL, and its efficacy has been widely recognized. Clinical trials have reported that CAR T therapy surpasses ASCT for refractory or early relapsed DLBCL. [26, 27] Consequently, current guidelines recommend CAR T therapy as the second-line treatment for such DLBCL cases. However, retrospective studies have shown that ASCT may yield lower relapse rates and higher OS compared to CAR T therapy in relapsed DLBCL patients who achieve partial remission with chemotherapy, including those with early relapsed or refractory disease. [28] Our results also showed high OS and PFS in the TT/BU regimen group, among patients with refractory disease or relapse within one year following first-line treatment.

ASCT has a long history of clinical trials and practical application, providing substantial long-term outcome data compared to the relatively newer CAR T therapy. Additionally, the management of side effects is well-established for ASCT, supported by years of experience. In contrast, CAR T therapy requires specialized knowledge to handle novel side effects, such as cytokine release syndrome and immune effector cell-associated neurotoxicity syndrome (ICANS).

Moreover, thiotepa-containing ASCT regimens have shown promise in treating DLBCL with central nervous system involvement, where the efficacy of CAR T therapy has not been properly established. This suggests that thiotepa-based ASCT regimens could be an effective therapeutic option alongside CAR T therapy, tailored to the individual patient’s condition and the characteristics of the lymphoma. Thus, for relapsed or refractory DLBCL, especially in cases sensitive to chemotherapy, ASCT remains a viable treatment option that may offer superior outcomes in specific scenarios, complementing the use of CAR T therapy in contemporary treatment strategies.

The limitations of this study include its retrospective design and the fact that it was conducted at a single center, which may limit the generalizability of the findings. Further large-scale, preferably prospective studies are warranted to validate the findings suggested in this study.

In conclusion, our retrospective analysis suggests that novel conditioning regimens like the TT/BU regimen offer a safer and potentially more effective option for patients with systemic DLBCL. Alongside CAR T therapy, which has its own advantages, TT/BU regimen-based ASCT remains an essential component of the evolving treatment paradigm for relapsed and refractory DLBCL.

## Supplementary Information

Below is the link to the electronic supplementary material.

Supplementary file1 (DOCX 250 KB)

Supplementary file2 (DOCX 242 KB)

## Data Availability

Raw data were generated at Kansai Electolic Power Hospital. Derived data supporting the findings of this study are available from the corresponding author K.Io on request.
