# Supplementary material for: Efficacy and safety of the thiotepa–busulfan conditioning regimen as for autologous stem cell transplantation in relapsed/refractory systemic diffuse large B cell lymphoma: a single-center retrospective study
Source: Int J Hematol. 2025 Feb 12;121(6):813–9. doi: 10.1007/s12185-025-03946-w (PMC12106555; doi:10.1007/s12185-025-03946-w)
Supplement: Supplementary file 1 — Supplementary file1 (DOCX 250 KB) [file 12185_2025_3946_MOESM1_ESM.docx]

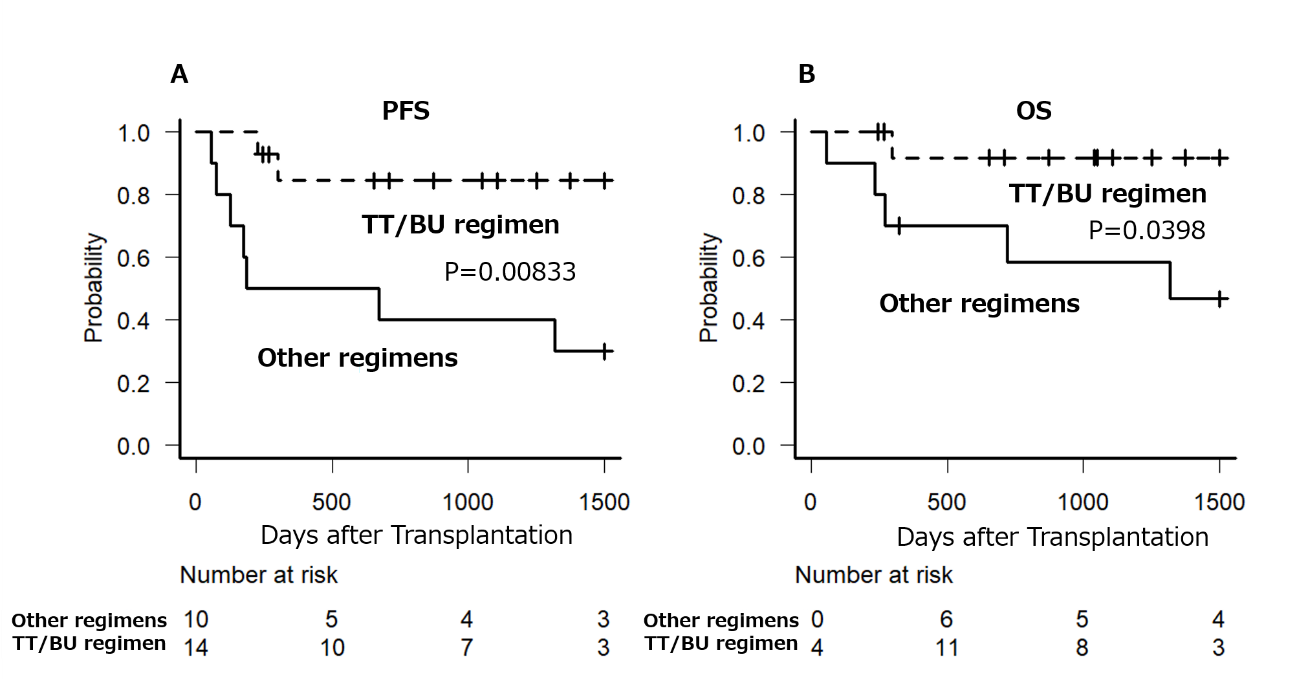


Supplementary Fig.1: Progression-free survival (A) and Overall survival (B) after autologous hematopoietic stem cell transplantation according to high-dose chemotherapy regimens　for patients in complete remission at the time of transplantation.

TT,thiotepa; BU,busulfan; P;p-value
